# Mechanism of Action of Small-Molecule Agents in Ongoing Clinical Trials for SARS-CoV-2: A Review

**DOI:** 10.3389/fphar.2022.840639

**Published:** 2022-02-25

**Authors:** Lei Zhao, Song Li, Wu Zhong

**Affiliations:** ^1^ National Engineering Research Center for the Emergency Drug, Beijing Institute of Pharmacology and Toxicology, Beijing, China; ^2^ Beijing Sunho Pharmaceutical Co., Ltd., Beijing, China

**Keywords:** mechanism of action, SARS-CoV-2, small-molecule drug, antiviral, COVID-19, clinical

## Abstract

Since the first reports from December 2019, COVID-19 caused an overwhelming global pandemic that has affected 223 countries, seriously endangering public health and creating an urgent need for effective drugs to treat SARS-CoV-2 infection. Currently, there is a lack of safe, effective, and specific therapeutic drugs for COVID-19, with mainly supportive and symptomatic treatments being administered to patients. The preferred option for responding to an outbreak of acute infectious disease is through drug repurposing, saving valuable time that would otherwise be lost in preclinical and clinical research, hastening clinical introduction, and lowering treatment costs. Alternatively, researchers seek to design and discover novel small-molecule candidate drugs targeting the key proteins in the life cycle of SARS-CoV-2 through an in-depth study of the infection mechanism, thus obtaining a number of candidate compounds with favorable antiviral effects in preclinical and clinical settings. There is an urgent need to further elucidate the efficacy and mechanism of action of potential anti-SARS-CoV-2 small-molecule drugs. Herein, we review the candidate small-molecule anti-SARS-CoV-2 drugs in ongoing clinical trials, with a major focus on their mechanisms of action in an attempt to provide useful insight for further research and development of small-molecule compounds against SARS-CoV-2 infection.

## 1 Introduction

Since its identification in December 2019, severe acute respiratory syndrome coronavirus 2 (SARS-CoV-2) has spread, resulting in a global pandemic of coronavirus disease 2019 (COVID-19), an acute respiratory infection posing a severe threat to human health and public health security on a global scale (Wang C. et al., 2020; Zhou et al., 2020). With the continuous spread of SARS-CoV-2, various new variants have emerged, the four most notable ones being alpha, beta, gamma, and delta, which have caused recent outbreaks in many countries and regions, thus representing a major challenge in the fight to curb the pandemic (Davies et al., 2021; Faria et al., 2021; World Health Organization, 2021). According to the most recent statistics from Johns Hopkins University, as of 4 November 2021, there are 248,625,178 confirmed cases of COVID-19 infection, which has claimed 5,029,325 lives across 223 countries worldwide (Coronavirus Resource Center, 2021).

In the face of the COVID-19 pandemic, scientists are actively engaged in researching and developing effective drugs against SARS-CoV-2. The preferred strategy in conditions of an acute infectious disease outbreak is to rapidly repurpose existing effective drugs, thus saving time for preclinical/clinical research and significantly reducing the time and cost associated with drug development (Wang M. et al., 2020; Talevi and Bellera, 2020). Alternatively, researchers have sought to design and discover novel small-molecule drugs targeting key proteins in the SARS-CoV-2 life cycle through an in-depth study of the mechanisms underlying infection, with a number of small-molecule candidate drugs already described (Dai et al., 2020; Yin et al., 2020). At present, only remdesivir and molnupiravir have been approved by the FDA and MHRA, respectively, for the treatment of COVID-19. Several candidate drugs have also shown promising antiviral efficacy in preclinical and clinical trials. Nevertheless, there is an urgent need to comprehensively elucidate the mechanism of action of these small-molecule drugs, identify potential drug targets, and develop more effective and safer anti-SARS-CoV-2 agents.

## 2 SARS-CoV-2: Biological Characteristics, Life Cycle, and Drug Targets

SARS-CoV-2 is a single-stranded, positive-sense, enveloped RNA virus, approximately 60–140 nm in diameter (Zhu N. et al., 2020). It belongs to the genus β-coronavirus in the Coronaviridae family, sharing 79% sequence homology with SARS-CoV, yet having a higher transmission capacity (Lu et al., 2020; Xu et al., 2020). The genome consists of approximately 29,900 bases and encodes 29 proteins, including 16 non-structural proteins (NSPs), with at least 13 downstream open-reading frames (ORFs) (Wu F. et al., 2020). Among NSPs, papain-like protease and 3C-like protease (3CLpro, NSP5) cleave pp1a and pp1b polypeptides into at least 16 NSPs. NSP7, NSP8, and NSP12 constitute RNA-dependent RNA polymerases responsible for SARS-CoV-2 replication and transcription. The downstream coding region mainly encodes structural proteins, including spike (S), membrane (M), envelope (E), nucleocapsid (N) protein, and several accessory proteins (Zhu G. et al., 2020).

The SARS-CoV-2 infection cycle is divided into several steps: attachment, entry (endocytosis and membrane fusion), RNA and protein synthesis, virion assembly, and release. As with most viruses, attachment to host cells is the first step initiating infection. The S protein receptor-binding domain interacts with host cell surface receptor proteins to facilitate attachment. The main host cell receptors involved are the angiotensin-converting enzyme 2 (ACE2), widely expressed in various tissues and organs, and the recently reported CD147-spike protein (Hoffmann et al., 2020a; Wang K. et al., 2020). After attachment, SARS-CoV-2 entry is achieved via endocytosis and/or direct membranes fusion, with S2 being cleaved by different proteases depending on the entry pathway. In the presence of TMPRSS2, the S protein undergoes proteolytic cleavage by host proteases TMPRSS2 and TMPRSS11D, allowing for viral-host membrane fusion. The SARS-CoV-2-ACE2 complex is internalized into endosomes via clathrin-mediated endocytosis in the absence of TMPRSS2, where S2 is cleaved by cathepsin L in the acidic environment to facilitate membrane fusion (Jackson et al., 2021). In both entry patterns, enzymatic cleavage discloses the fusion peptide and induces conformational changes in the S2 subunit, driving the peptide to move into the membrane and thus launching membrane fusion (Jackson et al., 2021). Genomic nucleic acid is then released into the cytoplasm for the following replication and translation. The genomic RNA acts as a template and attaches to ribosomes to translate viral proteases and polymeric protein precursors, which are then cleaved by 3CLpro and PLpro, respectively, to form functional NSPs. NSP12, NSP7, and NSP8 form the SARS-CoV-2 RNA-dependent RNA polymerase (RdRp) complex, responsible for the replication and transcription of genomic RNA in the cytoplasm (Gao et al., 2020). At the same time, the subgenomic mRNA is translated into structural and accessory proteins, utilizing the host translation machinery. In the assembly and release stage, the nucleocapsid, formed through the assembly of genomic RNA and the N protein, assembles with envelope proteins to then form progeny virions (Chen et al., 2020). Finally, the virions are released from infected cells to continue the viral replication cycle.

**FIGURE 1:**
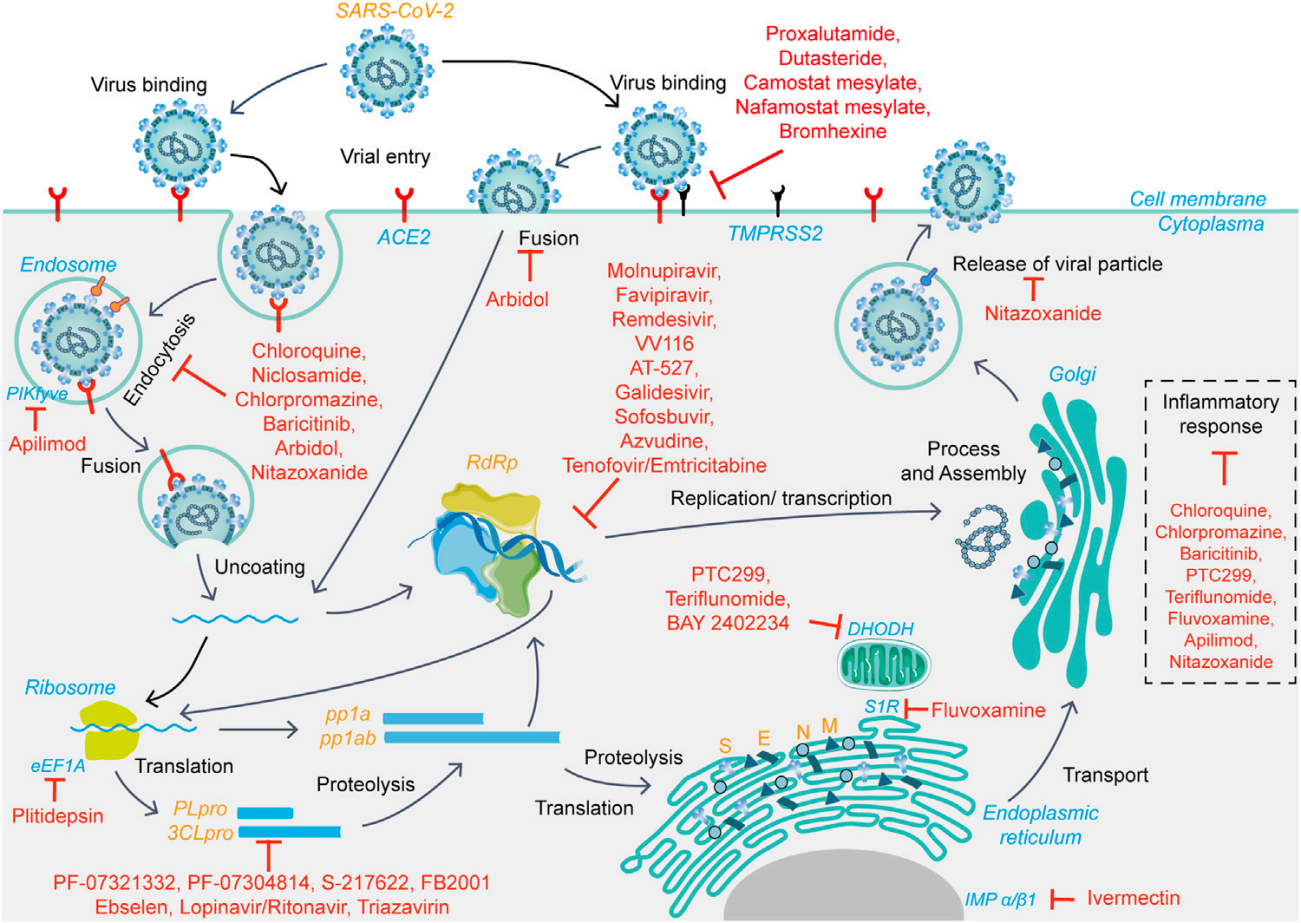
The viral life cycle of SARS-CoV-2 and potential mechanisms of action of small-molecule candidate drugs.

Therefore, drugs targeting viral proteins related to the virus life cycle (Figure 1), including but not limited to 3CLpro, PLpro, and RdRp, as well as host-related target proteins, such as ACE2 and TMPRSS2, to block virus entry and replication, are expected to have therapeutic potential.

## 3 Small-Molecule Agents in Ongoing Clinical Trials for COVID-19

Although the SARS-CoV-2 pandemic has slowed to some extent due to widespread vaccination, there is an urgent need for effective antiviral drugs. The results of the Solidarity Clinical Trial, a large-scale, randomized clinical study carried out by WHO, indicated that the four candidate drugs identified early in the outbreak (such as remdesivir) are ineffective or have little effect on COVID-19 (Pan et al., 2021). Antibody formulations and other therapies have not made satisfactory clinical progress either. Small-molecule oral agents have obvious advantages with regard to delivery and availability, with potential antiviral activity against mutant strains, highlighting their potential role in the fight against SARS-CoV-2. In addition to the FDA- and MHRA-approved remdesivir and molnupiravir, a total of 11 drugs and biologicals are authorized for emergency use, of which only baricitinib is a small-molecule drug (Food and Drug Administration, 2021). There are 20 approved drugs for COVID-19 worldwide, and more than 380 candidate drugs are undergoing clinical development. In the present work, we review small-molecule agents at the clinical stage or approved drugs (as shown in Table 1) as well as their respective mechanisms of action against SARS-CoV-2. The discussed agents include, but are not limited to, the highly touted molnupiravir, AT-527, PF-07321332, and S-217622.

**TABLE 1:** General information of anti-SARS-CoV-2 drugs in clinical development.

Drugs	No. of clinical trials registered^a^	Phase	Molecular target	Development strategy	Approval status(for COVID-19)
Remdesivir	77	4	RdRp	Repurposing	Approval by FDA
Favipiravir	46	4	RdRp	Repurposing	EUA in several countries
Molnupiravir	5	3	RdRp	Novel	Approval by MHRA; EUA by FDA
AT-527	3	3	RdRp	Novel	Non-approved
Galidesivir	1	1	RdRp	Repurposing	Non-approved
Sofosbuvir	8	4	RdRp	Repurposing	Non-approved
Azvudine	3	3	RdRp	Repurposing	Non-approved
Tenofovir/emtricitabine	5	3	RdRp	Repurposing	Non-approved
PF-07321332	8	3	3CLpro	Novel	EUA by FDA
PF-07304814	3	1	3CLpro	Novel	Non-approved
s-217622	—	2/3	3CLpro	Novel	Non-approved
FB2001	1	2/3	3CLpro	Novel	Non-approved
Ebselen	2	2	3CLpro	Repurposing	Non-approved
Lopinavir/ritonavir	24	4	3CLpro	Repurposing	Non-approved
Triazavirin	2	4	RNA synthesis/3CLpro	Repurposing	Non-approved
Chloroquine/hydroxychloroquine	46/276	4	Endosomal entry	Repurposing	EUA by FDA at earlier outbreak (chloroquine)
Umifenovir/arbidol	3	4	Endosomal entry	Repurposing	Non-approved
Niclosamide	11	3	Endosomal entry	Repurposing	Non-approved
Chlorpromazine	2	3	Endosomal entry	Repurposing	Non-approved
Baricitinib	20	4	Endosomal entry	Repurposing	EUA by FDA
Proxalutamide	5	3	Androgen receptor antagonist	Repurposing	Non-approved
Dutasteride	1	2	5-alpha-reductase inhibitor	Repurposing	Non-approved
Camostat mesylate	5	3	TMPRSS2 inhibitor	Repurposing	Non-approved
Nafamostat mesylate	2	2	TMPRSS2 inhibitor	Repurposing	Non-approved
PTC299	1	2	DHODH inhibitor	Repurposing	Non-approved
Teriflunomide	3	3	DHODH inhibitor	Repurposing	Non-approved
Nitazoxanide	23	4	Endosomal entry/Inflammatory response regulation	Repurposing	Non-approved
Fluvoxamine	1	3	Sigma-1 receptors agonist	Repurposing	Non-approved
Plitidepsin	3	3	eEF1A inhibitor	Repurposing	Non-approved
Ivermectin	69	4	IMPA/β1 inhibitor	Repurposing	Non-approved
Apilimod	1	2	PIKFYVE inhibitor	Repurposing	Non-approved

a Registered on ClinicalTrials.gov.

## 4 Mechanism of Action of Small-Molecule Agents

### 4.1 Antiviral Agents Targeting Viral Proteins

#### 4.1.1 Inhibitors of SARS-CoV-2 RdRp

Remdesivir is a nucleoside analog developed by Gilead, with inhibitory effects against Ebola, MERS, and SARS viruses. The EC_50_ of remdesivir against SARS-CoV-2 was reported to be 0.77 μM in Vero E6 cells (Wang X. et al., 2020). As a prodrug, remdesivir is converted to a triphosphate derivative by host enzymes. It then competitively binds the RdRp of SARS-CoV-2, leading to its incorporation into the growing viral RNA chain and the premature termination of chain synthesis, that is, the inhibition of viral replication (Yin et al., 2020). Remdesivir is the first FDA-approved drug for the treatment of COVID-19, but it has become controversial with increasing clinical evidence questioning its efficacy. The poor *in vivo* efficacy of remdesivir may be attributed to the design based on hepatic targeting and the rate of conversion to the active form. Remdesivir is a phospholipid amide prodrug with the parent nucleoside GS-441524, which is designed for hepatic targeting and can be rapidly metabolized and converted in such cells. In contrast, the target cells of SARS-CoV-2 are mainly respiratory and lung-associated cells, which may not sufficiently metabolize the drug. In addition, remdesivir is converted to GS-441524 in plasma, and its initial conversion to monophosphate form is slow and considered as the rate-limiting step. VV116 is a novel nucleoside analog that is modified with remdesivir as a prototype, primarily through a prodrug strategy to improve its oral bioavailability and anti-SARS-CoV-2 activity. The anti-SARS-CoV-2 EC_50_ of VV116 was 0.35 μM compared to 1.71 μM for remdesivir in Vero E6 cells, and VV116 was also effective in reducing lung viral load and titer in a dose-dependent manner in a mouse infection model (Xie et al., 2021). Based on the preclinical results, the drug is in phase I clinical trials for the treatment of COVID-19 in Uzbekistan.

Favipiravir is a nucleoside analog developed by Toyama Chemical Co. Ltd., used to treat influenza in Japan and China. Favipiravir is a broad-spectrum antiviral drug, including influenza, arboviruses, filoviruses, bunyavirus, and coronaviruses. Favipiravir was reported to have an EC_50_ of 61.88 μM against SARS-CoV-2 in Vero E6 cells (Wang M. et al., 2020). Several clinical trials have preliminarily demonstrated its efficacy and safety (Cai et al., 2020; Fu et al., 2020; Ivashchenko et al., 2021), with large-scale clinical trials currently underway (Appili Therapeutics Inc., 2020; PRINCIPLE, 2021). Favipiravir has been authorized for emergency COVID-19 treatment in several countries, such as Turkey, India, and Russia. Favipiravir is a prodrug and its active form, favipiravir ribofuranosyl-triphosphate, inhibits viral replication by targeting RdRp. There are two main hypotheses regarding the action of favipiravir at present (Zhao and Zhong, 2021). Naydenova et al. reported that favipiravir ribofuranosyl-triphosphate, as a nucleoside analog, simulates guanosine triphosphate (GTP) or adenosine triphosphate (ATP) to be mixed into the RNA of nascent viruses by the SARS-CoV-2 RdRp complex, inducing the termination of RNA synthesis (Naydenova et al., 2021). In contrast, several studies based on the structural analysis of viral RdRp-drug complexes showed that favipiravir ribofuranosyl-triphosphate mimicked the incorporation of ATP and GTP nucleotides into nascent RNA products and induced high rates of genomic mutation that led to the production of non-viable virions, further inhibiting viral replication and reproduction (Shannon et al., 2020; Peng et al., 2021; Zhao and Zhong, 2021). In the presence of intracellular ATP or GTP, the termination of RNA synthesis is unlikely. Favipiravir and remdesivir have different antiviral mechanisms, which may be due to the differences in their influence on RdRp, although they both inhibit its activity.

Molnupiravir (EIDD-2801) is an isopropyl prodrug of the nucleoside analog β-D-N4-hydroxycytidine that targets the RdRp. It displays a broad-spectrum antiviral activity, including influenza, Ebola, and coronaviruses. The IC_50_ of molnupiravir against SARS-CoV-2 was 0.3 and 0.08 μM in Vero and Calu-3 cells, respectively, with a CC_50_ >10 μM. Molnupiravir effectively prevents and treats SARS-CoV-2, SARS-CoV, and MERS-CoV infections in mice (Sheahan et al., 2020; Wahl et al., 2021). In ferret models, molnupiravir effectively reduced viral load and completely prevented virus transmission through direct contact with untreated animals (Cox et al., 2021). Several clinical trials have been conducted on molnupiravir as a potential agent for COVID-19 treatment. In April 2021, Merck announced discontinuing studies in hospitalized patients due to a lack of efficacy. In early October and November, Merck and Ridgeback Biotherapeutics announced interim data and additional analyses, respectively, from a clinical trial (NCT04575597, MOVe-OUT) of molnupiravir for the treatment of patients with mild or moderate COVID-19. In the analysis, 6.8% of molnupiravir-treated patients were either hospitalized or died (48/709) following randomization, compared with 9.7% of patients who received placebo (68/699) through day 29 (*p* = 0.0218). Molnupiravir reduced the risk of hospitalization or death by approximately 30% (Merck, 2021a). The MHRA has granted authorization for molnupiravir in the United Kingdom, making it the first oral antiviral drug for treating adult patients with mild or moderate COVID-19 and at least one risk factor for developing severe illness (Merck, 2021b).

According to systematic biochemical and structural analyses, Kabinger et al. (2021) suggested that molnupiravir exerts its antiviral effects by inducing SARS-CoV-2 RNA mutagenesis and proposed a two-step model. NHC triphosphate, the active form of molnupiravir, can be misidentified by the RdRp of SARS-CoV-2 as CTP or UTP during RNA synthesis. Therefore, when the positive-strand genomic RNA (+gRNA) is used by SARS-CoV-2 RdRp as a template to synthesize subgenomic RNA (sgRNA) and negative-strand gRNA, in the first step, M is incorporated into sequences instead of U or C. In the next step, the resulting M-containing negative-strand gRNA is used as a template for synthesizing +gRNA, which results in mutations in these newly synthesized RNAs. This mutagenesis of RNA is predicted not to maintain the formation of intact progeny viruses. This conclusion is consistent with previous results on the mechanism of molnupiravir against other viruses. Taken together, molnupiravir, as a mutagen, causes “error disasters” during viral replication, thereby exerting an antiviral effect (Sheahan et al., 2020; Toots et al., 2020). These results also suggest that molnupiravir-induced mutagenesis could be the molecular mechanism for its broad-spectrum anti-RNA virus activity. It is important to note that a study showed that molnupiravir could introduce mutations not only in the genome of SARS-CoV-2 but also in mammalian cells (Zhou et al., 2021). It is not clear whether it causes mutations in humans. In animal studies and clinical trials led by Merck, no mutagenic potential was found at normal doses in the duration of therapy. Therefore, the drug should be strictly administered as prescribed regarding dosing and contraindications such as pregnancy, and its risk should be evaluated over time.

AT-527, the hemisulfate form of AT-511, is an orally available purine nucleotide analog prodrug co-developed by Roche and Atea, which has pan-genotypic anti-HCV replication activity (Good et al., 2020). AT-527 is converted to a free base upon entry into cells and is subsequently metabolized to the triphosphate form metabolite AT-9010. AT-511 inhibited SARS-CoV-2 replication with an EC_90_ of 0.47 μM in human respiratory epithelial cells, with similar activity against coronaviruses in Huh7 cells (Good et al., 2021). Of note, recent studies reported no anti-SARS-CoV-2 activities for AT-511 in a variety of cell models, including Vero, Huh7, and human respiratory epithelial cells. The author suggests that differences between models may contribute to differences in the metabolism of AT-511 into the active form, thus compromising its antiviral activity (Do et al., 2021). Since AT-511 is a double prodrug requiring multiple enzymes for activation, it may be more susceptible to different experimental conditions (Do et al., 2021). To explore the mechanism of action of AT-527/AT-511 against SARS-CoV-2, Canard et al. (2021) determined the structure of the SARS-CoV-2 RdRp:RNA:AT-9010 complex via cryoelectron microscopy reconstruction and identified three sites of AT-9010 binding to NSP12. The compound is inserted into the nascent RNA strand at the activation site, and its 2'-methyl group blocks the accurate positioning of the second AT-9010 inserted into the following NTP site. Therefore, the incorporation of AT-9010 terminates the RNA strand extension, and the subsequent AT-9010 disrupts the catalytic site of the complex, which exhibited resistance to ExoN removal. Besides, the 5'-diphosphate of AT-9010 stably binds to the specific NSP12 N-terminal NiRAN domain of SARS-CoV-2, a structural and functional homolog of selenoprotein-O pseudokinase. This unique binding mode blocks the NiRAN-mediated UMPylation of SARS-CoV-2 NSP8 and NSP9 (Canard et al., 2021). The results indicate that AT-9010, the active form of AT-527, may exert anti-SARS-CoV-2 activity through a dual mechanism of inhibition via binding to both RdRp and NiRAN active sites. Atea evaluated AT-527 across multiple clinical trials, including the phase II MOONSONG virology study in patients with mild or moderate COVID-19, a global phase II study in hospitalized patients with moderate COVID-19, and the global phase III MORNINGSKY trial. On 19 October 2021, Atea Pharmaceuticals Inc. (2021) reported that the MOONSONG trial failed to meet its primary endpoint of reducing the viral load in patients treated with AT-527 relative to placebo in the overall population; however, it appeared to be effective in high-risk patients with underlying health conditions. The phase III MORNINGSKY trial (NCT04889040) is currently underway and will be completed in the second half of 2022.

Galidesivir (BCX4430), an adenine analog originally developed by BioCryst to treat HCV, targets viral RdRp. Upon entry into cells, galidesivir is converted by host kinases to its triphosphate derivative, which is capable of mimicking ATP and is inserted by the RdRp complex into the newly synthesized RNA strand, inducing premature termination of nascent RNA (Taylor et al., 2016). Regarding the interaction of SARS-CoV-2 RdRp and galidesivir, docking analyses using the CCDC GOLD program demonstrated that galidesivir established six hydrogen and four hydrophobic bond interactions with NSP12, with an important one being the bond interaction with the catalytic center Asp^760^ or Asp^761^(Zhang W. F. et al., 2020). In addition, interactions between galidesivir and Asp^760^/Asp^761^/Asp^618^ may interfere with the chelation of metal ions, thus affecting the activity of RdRp. Some studies have shown that galidesivir has broad-spectrum antiviral activity against filoviruses, paramyxoviruses, coronaviruses, and SARS-CoV-2 *in vitro*, with an EC_50_ ranging from 3 to 68 μM (Taylor et al., 2016; Dömling and Gao, 2020). A phase II clinical trial of galidesivir against COVID-19 is currently underway.

Sofosbuvir is a pyrimidine nucleotide analog approved by the FDA for the treatment of HCV. Sofosbuvir is effective against a variety of RNA viruses, including anti-SARS-CoV-2 activity in Huh7 cells, Calu-3 cells, and brain organoid models (Mesci et al., 2020; Sacramento et al., 2021). Several clinical trials on the efficacy of sofosbuvir/daclatasvir in patients with COVID-19 are underway in Iran (Sadeghi et al., 2020; Roozbeh et al., 2021). Sofosbuvir is converted to its active triphosphate form by cellular enzymes, and the active form is inserted into the newly synthesized RNA strand as a substrate of the SARS-Co-2 RdRp, immediately blocking the further extension of the RNA strand owing to the fluoro and methyl modifications at the 2' position, thereby inhibiting viral replication. Compared with natural nucleotide UMP excision, sofosbuvir terminated RNA was highly resistant to excision by the SARS-CoV-2 exonuclease complex (Jockusch et al., 2020b).

Azvudine is a novel nucleoside analog that was initially developed for HIV treatment. It is the first nucleoside analog to simultaneously inhibit nucleoside reverse transcriptase and restore the expression of cytosine deaminase APOBEC3G in HIV-1 patient-derived CD4^+^ T cells (Yu and Chang, 2020). In July 2021, the drug was approved as a class 1 new drug to treat HIV infection. Currently, several clinical trials have been conducted on azvudine for COVID-19. Yu and Chang (2020) suggested that the mechanism of azvudine against SARS-CoV-2 may be similar to that of remdesivir. After conversion to triphosphate *in vivo*, RdRp incorporates it into the nascent RNA strand, preventing the subsequent addition of nucleotides. Nascent RNA synthesis is terminated, thereby inhibiting viral replication. The preliminary results of a clinical trial showed that azvudine treatment might shorten the nucleic acid negative conversion time in patients with mild or common COVID-19 (2.6 vs. 5.6 days, *p* = 0.008) (Ren et al., 2020).

Tenofovir/Emtricitabine is an approved drug combination for the treatment and prevention of HIV infection, with more than nine clinical studies for treating COVID-19 currently registered on ClinicalTrials.gov. Mechanistically, tenofovir is phosphorylated to an active intracellular metabolite, and tenofovir triphosphate and emtricitabine triphosphate act on HIV reverse transcriptase, causing termination of viral DNA strand synthesis. The tenofovir/emtricitabine triphosphate binds tightly to SARS-CoV-2 RdRp and is incorporated as a substrate into the nascent RNA strand, resulting in the termination of strand synthesis (Jockusch et al., 2020a; Elfiky, 2020).

**FIGURE 2:**
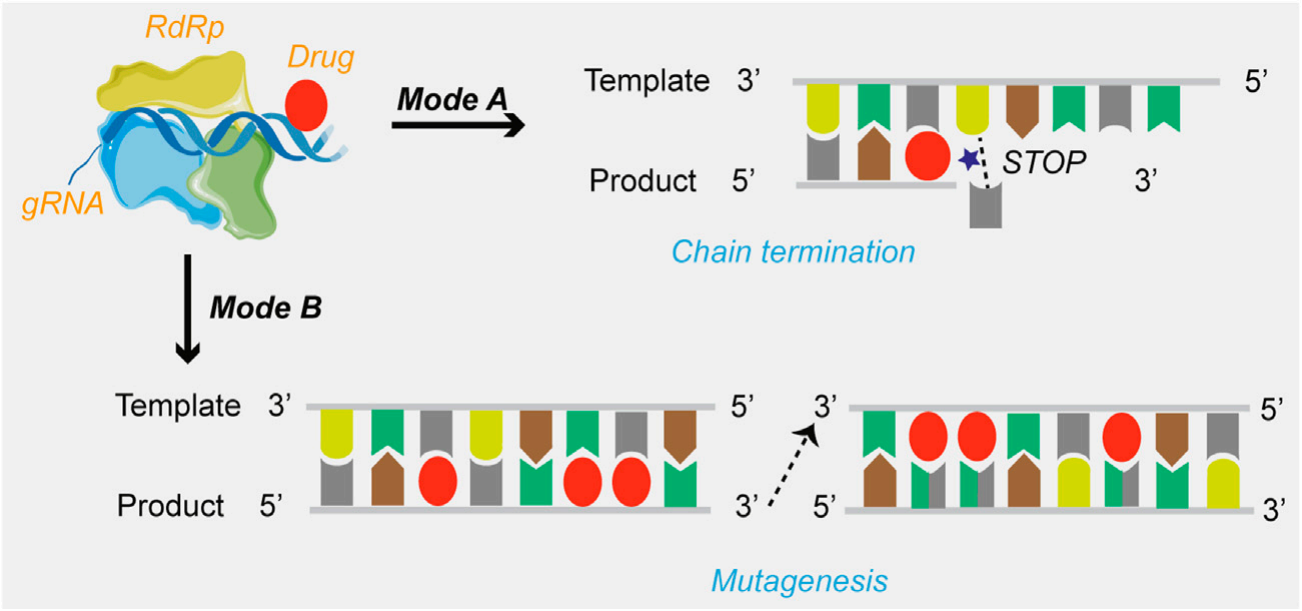
The mechanisms of drugs targeting SARS-CoV-2 RdRp. Mode A: Chain termination; Mode B: Viral RNA mutagenesis induction.

Currently, drug candidates targeting viral RdRp are the most dominant class in clinical trials for the treatment of COVID-19. Based on the review of the mechanisms of action, the mechanisms of SARS-CoV-2 RdRp complex inhibition can be grouped into two modes (Figure 2): immediate chain termination (e.g., remdesivir, galidesivir, sofosbuvir, and azvudine) and induced mutagenesis (e.g., favipiravir and molnupiravir). The difference in the mechanism may be attributed to differences in the structural complexity of the heterocycle of small molecules.

#### 4.1.2 Inhibitors of Viral Proteases-3CLpro

PF-07321332 is an orally available inhibitor of 3CLpro, which plays a major role in the life cycle of coronaviruses. On 5 November 2021, Pfizer announced an interim analysis of phase II/III randomized, double-blind study of PAXLOVID™ in non-hospitalized adult patients with COVID-19, who are at high risk of progressing to severe illness. PAXLOVID™, a combination of PF-07321332 and ritonavir, significantly reduced hospitalization and death from any cause (Pfizer Inc., 2021). Further, 0.8% (3/389) of patients were admitted to the hospital through day 28 with no deaths in the PAXLOVID™ group, compared to 7.0% (27/385) of patients were admitted, with seven subsequent deaths in the placebo group. An 89% reduction in the risk of COVID-19 hospitalization or death was reported in patients with PAXLOVID™ treatment within 3 days of symptom onset (*p* < 0.0001). Regarding safety, 19 and 21% of patients experienced adverse events in the PAXLOVID™ and placebo group, respectively, with the former being less likely to report serious adverse events (1.7 vs. 6.6%) (Mahase, 2021). Pfizer will cease further enrollment and plans to submit the data to the FDA for Emergency Use Authorization (EUA). As a 3CLpro inhibitor, PF-07321332 reacts with a cysteine residue at the protease-binding site to prevent viral replication in cells. This cysteine plays a key role in the 3CLpro activity of coronaviruses. Therefore, activity inhibition prevents the viral genome from encoding functional NSPs, ultimately suppressing viral replication (Muramatsu et al., 2016; Ionescu, 2020). PF-07321332, which contains a nitrile group, is a polypeptide covalent inhibitor. As reversible site-specific inhibitors, peptide nitriles react with catalytic cysteine to form sulfides. The inhibitor presents a g-lactam ring, a common feature of most covalent inhibitors developed against SARS-CoV-2 3CLpro, which exploits the selectivity of glutamine residues at cleaved multiprotein equivalent sites (Ramos-Guzmán et al., 2021). Ramos-Guzmán et al. revealed some critical characteristics of PF-07321332 using computational simulations. The inhibitor acts in two stages: forming a non-valent complex with the enzyme and a reaction with the catalytic cysteine to form sulfide (Ramos-Guzmán et al., 2021). With regard to the binding process, molecular dynamics simulations and free energy calculations indicated that most of the affinity between the inhibitor and the enzyme was due to the P1 and P2 groups, which are identical or similar to most known inhibitors of this protease; While the P3 and P4 groups also contribute to the binding process, the free energy contribution is much smaller, suggesting that it is still possible to improve the affinity of 3CLpro (Ramos-Guzmán et al., 2021). The low molecular weight of the nitrile group appears to be superior to covalent inhibition. The resulting sulfide is steadier than the initial non-covalent complex, indicating that the inhibition is more irreversible compared to that of aldehydes or ketones derivatives.

PF-07304814, an early intravenously administered drug developed by Pfizer, is also a 3CLpro inhibitor and is hydrolyzed into its active form, PF-00835231. Recent studies have shown that PF-07304814 also has inhibitory activity against the 3CLpro of SARS-COV-2. In Vero E6 cells, the EC_50_ of PF-07304814 against SARS-CoV-2 was 40 μM, and, after the addition of p-glycoprotein, the EC_50_ was 0.48 μM (Boras et al., 2021). Currently, the drug is in phase I clinical trials (NCT04627532 and NCT04535167) for COVID-19 treatment.

S-217622 is a small-molecule anti-SARS-CoV-2 candidate drug developed by Shionogi, Japan, which inhibits 3CLpro activity to prevent viral infection. S-217622 exhibited *in vitro* antiviral activity against multiple SARS-CoV-2 strains, including alpha, beta, gamma, and delta. In an animal model, the viral load was rapidly and significantly reduced after administration (Shionogi & Co., Ltd., 2021a). Phase I clinical trials conducted in July 2021 in Japan showed that oral administration of S-217622 was safe, with once-daily oral dosing reducing plasma concentrations, as predicted from preclinical studies (Shionogi & Co., Ltd., 2021c). Phase II/III clinical trials for patients with mild COVID-19 or asymptomatic infection are currently underway (Shionogi & Co., Ltd., 2021b).

FB2001 is a protease inhibitor developed by the Shanghai Institute of Materia Medica, Chinese Academy of Sciences. The antiviral drug was designed and synthesized based on the three-dimensional structure of SARS-CoV-2 3CLpro with the aim of effectively blocking viral replication and inhibiting progeny virions production. At the cellular level, the EC_50_ of FB2001 against SARS-CoV-2 was 0.53 μM, and it showed better pharmacokinetic properties and safety in rat and beagle dog models (Dai et al., 2020). In terms of its inhibitory mechanism on 3CLpro of SARS-CoV-2, a structure-based study revealed the FB2001-3CLpro binding mode. FB2001 occupied the substrate-binding pocket and further inhibited 3CLpro activity. The catalytic site Cys^145^ of 3CLpro forms a covalent bond with the carbon atom of the aldehyde group of FB2001, and the Oxygen atom of the aldehyde group stabilizes the compound conformation by forming a hydrogen bond with Cys^145^ at the S1' site. The oxygen atom of the lactam group forms a hydrogen bond with the side chain of His^163^, the NH group also forms hydrogen bonds with Phe^140^ and Glu^166^, stabilizing the lactam group. In addition, the cyclohexyl group of the FB2001 interacts with the side chains of residues Asp^187^, Arg^188^, Met^49^, Met^165^, and Tyr^54^ through hydrophobic interactions, and the indole group interacts with residue Gln^189^ and Pro^168^ in the same way (Dai et al., 2020). FB2001 has been initiated in a phase I/II clinical trial (NCT04766931) in the United States in March 2021 to evaluate the safety and efficacy of FB2001 for the treatment of COVID-19.

Ebselen is a small-molecule organoselenium compound with glutathione peroxidase activity. The IC_50_ of its anti-SARS-CoV-2 activity in Vero cells was 4.67 μM, and the EC_50_ of its hydrolytic activity against 3CLpro of SARS-CoV-2 was 0.67 μM, suggesting that ebselen may exert antiviral effects by inhibiting SARS-CoV-2 3CLpro (Jin et al., 2020). Amporndanai et al. investigated the binding mode of 3CLpro and ebselen via high-resolution co-crystallography. They found that the formation of a selenyl sulfide bond between Cys^145^ of the catalytic dyad and the free selenium atom at the catalytic site inhibited the activity of 3CLpro. Further, this binding did not impact the conformational changes of amino acid disability around the active site. Combined with the LC/MS method, it was shown that after ebselen hydrolysis, the selenium atom binds directly to the catalytic site Cys^145^ to exert antiviral effects (Amporndanai et al., 2021). Phase II clinical trials of ebselen for treating patients with moderate to severe COVID-19 are currently ongoing.

Both ritonavir and lopinavir are HIV protease inhibitors. Ritonavir inhibits aspartic protease, which prevents processing of the Gag-Pol polyprotein precursor, and lopinavir inhibits Gag-Pol polyprotein division. They act synergistically to reduce the viral load by disabling the regeneration of viral particles (Cvetkovic and Goa, 2003). They are likely to exert antiviral activity by inhibiting 3CLpro of coronaviruses, including SARS-CoV-2 (Wu et al., 2004; Uzunova et al., 2020). A clinical study showed that lopinavir/ritonavir was less effective than favipiravir in terms of viral clearance and disease progression, with more adverse events recorded (Cai et al., 2020). In addition, several clinical trials, including Solidarity conducted by the WHO, indicated that lopinavir/ritonavir did not significantly reduce mortality and clinical symptoms of patients with COVID-19 (Cao et al., 2020; Pan et al., 2021).

Triazavirin is a guanine nucleotide analog that was originally approved for the treatment of influenza in Russia. Triazavirin has broad-spectrum antiviral activity against RSV, influenza, parainfluenza virus, and adenovirus, but its antiviral mechanism remains unclear. It is widely speculated to act as a purine nucleotide analog inhibiting viral RNA synthesis and replication of viral genome fragments (Wu X. et al., 2020). A computational docking study speculated that triazavirin might exert anti-SARS-CoV-2 effects as a 3CLpro inhibitor (Shahab and Sheikhi, 2020). Preliminary results from a randomized controlled trial of triazavirin for COVID-19 showed no significant benefit for patients with COVID-19. However, patients in this arm less frequently used concomitant therapies for hepatic, respiratory, cardiac, or renal conditions (Wu X. et al., 2020). Two clinical trials of triazavirin for the treatment of COVID-19 are ongoing in South Africa and Egypt.

**FIGURE 3:**
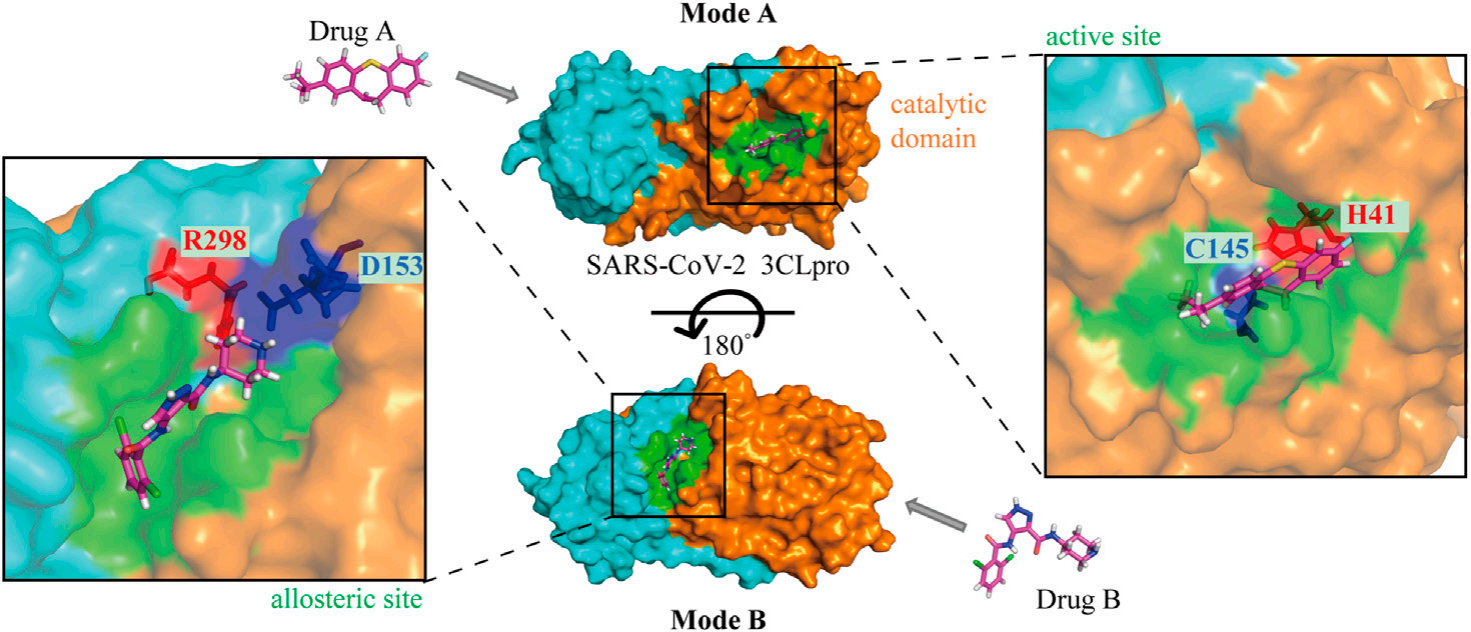
The mechanisms of drugs targeting SARS-CoV-2 3CLpro. Mode A: Binding to the active site of the catalytic domain; Mode B: Binding to the allosteric site. The orange and aqua areas represent the catalytic and dimerization domains, respectively. Drug A in the 3CLpro active site with catalytic residues labeled, and drug B in the allosteric site with residues labeled.

3CLpro is an ideal anti-SARS-CoV-2 target along with the classical target, RdRp. Drug repurposing screening and structure-based design of novel drug candidates targeting 3CLpro have become hot spots for anti-SARS-CoV-2 drug development since the cryo-electron microscopy structure has been reported. In general, the enzymatic activity of 3CLpro is dependent on the structure of the catalytic site, so the majority of 3CLpro inhibitors hinder the activity by affecting the dimerization of the enzyme and altering the structure of the catalytic active site in the substrate-binding pocket, especially the residue Cys^145^ at the S1’ site (Figure 3). In addition to the catalytic active site, the allosteric site may also be a binding region for drug interaction (Figure 3), although fewer compounds have been identified to act at this site. Further study on the key allosteric sites for enzyme inhibition may improve the efficiency of novel drug development.

### 4.2 Antiviral Agents Targeting Host Factors

#### 4.2.1 Inhibitors of Endosomal Entry

**FIGURE 4:**
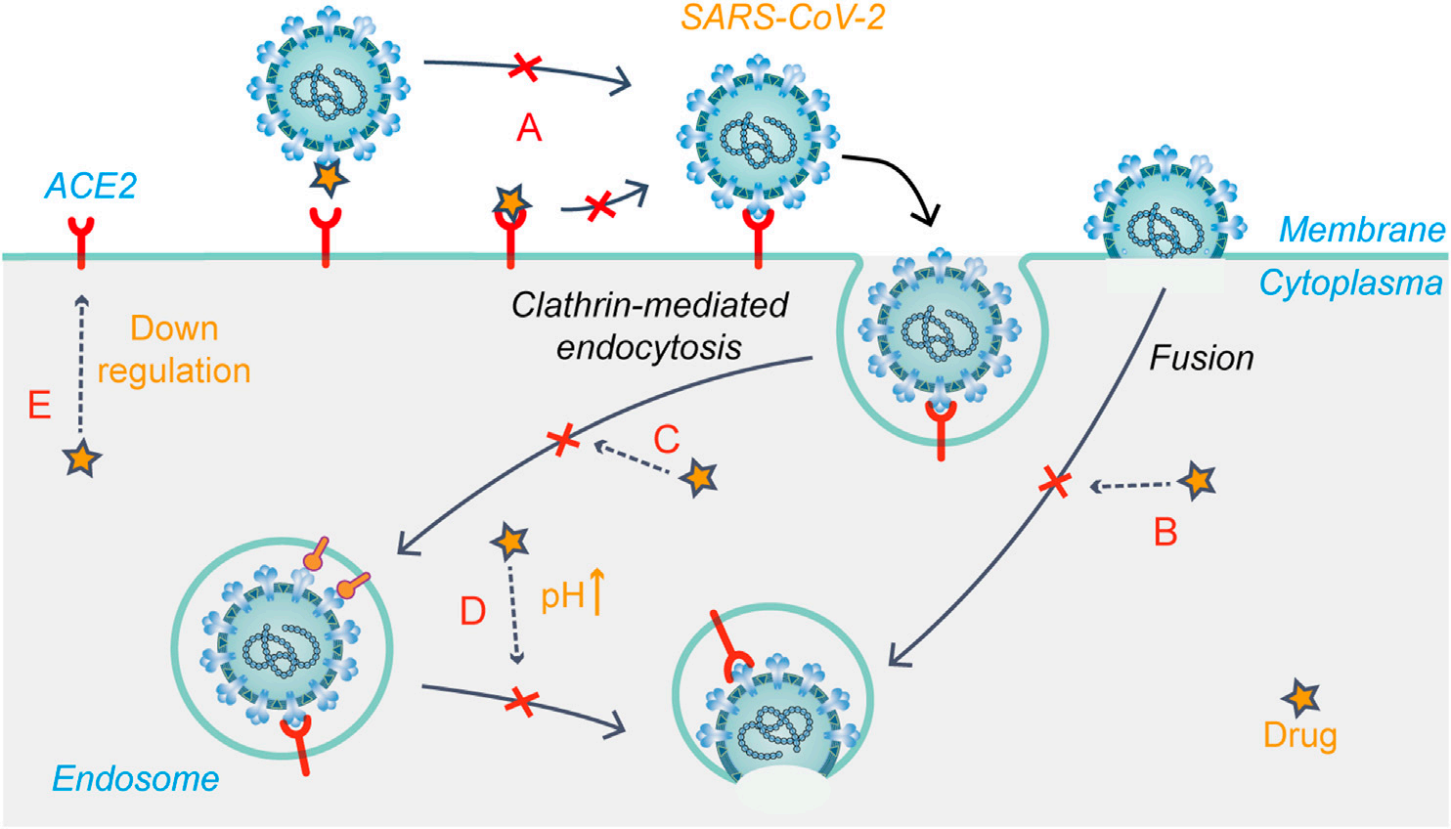
Schematic presentation of the possible mechanism that drug candidates target at early stages of SARS-CoV-2 life cycle. **(A)** Modification of S glycoprotein and the ACE2 receptor glycoprotein; **(B)** Fusion and release **(C)** Endocytosis-mediated viral entry; **(D)** Changing the acidic environment; **(E)** ACE2 receptor regulation.

Chloroquine was originally used to treat malaria, but its use declined with the emergence of drug-resistant malaria parasites. Chloroquine and hydroxychloroquine are also used for the treatment of rheumatic diseases because of their immunomodulatory effects. They have good inhibitory effects on a variety of coronaviruses, including SARS-CoV-2 (Rolain et al., 2007; Wang M. et al., 2020; Persoons et al., 2021). Based on relevant reports, the mechanism of action of chloroquine/hydroxychloroquine against coronavirus mainly involves the modification of viral S glycoprotein and post-translational modification of the ACE2 receptor glycoprotein (Li et al., 2003; Vincent et al., 2005). Chloroquine/hydroxychloroquine may inhibit SARS-CoV-2 replication through a variety of mechanisms, acting on both the virus and host cells. Studies have shown that chloroquine/hydroxychloroquine not only inhibits the endocytosis-mediated viral entry (Figures 4A, C) into host cells through its effect on endosome maturation but also affects the post-entry stage (Liu et al., 2020). Chloroquine phosphate effectively inhibited SARS-CoV-2 infection *in vitro*, shortened the time of viral clearance, and improved clinical symptoms in patients with COVID-19 (Huang et al., 2020). Early into the COVID-19 outbreak, the FDA granted chloroquine emergency authorization for the treatment of COVID-19. Since then, several clinical trials have shown that hydroxychloroquine is ineffective in COVID-19 and has serious side effects (Geleris et al., 2020; National Institutes of Health, 2020). Further, researchers negated the anti-SARS-CoV-2 activity of hydroxychloroquine in a human lung cell model (Hoffmann et al., 2020b). At present, hydroxychloroquine is considered ineffective against COVID-19, while the efficacy of chloroquine remains controversial.

Arbidol (umifenovir) could effectively inhibit viral fusion with host cells and has been approved to prevent and treat influenza in Russia. Previous studies have shown that arbidol effectively inhibits SARS-CoV-2 infection *in vitro* with an EC_50_ value of 4.11 μM. This study further investigated the action stages of arbidol on the virus life cycle, showing that the drug blocks SARS-CoV-2 entry by preventing attachment and release from intracellular vesicles (Wang X. et al., 2020). Molecular dynamics and structural studies suggested that there may be structural similarities between the drug-binding regions of influenza virus HA and SARS-CoV-2 S glycoprotein. Thus, arbidol may target the latter and impede its trimerization to exert antiviral effects (Vankadari, 2020) (Figures 4A,B). A meta-analysis showed that arbidol use was not related to symptom alleviation, nucleic acid negative conversion time, or mortality in COVID-19 patients compared to the control treatment (Huang et al., 2021). There is no clear evidence to support the efficacy of arbidol in treating COVID-19.

Niclosamide is an FDA-approved anthelmintic drug, which showed high anti-SARS-CoV-2 activity in Vero E6 cells and respiratory tract infection models (Jeon et al., 2020). The anti-SARS-CoV-2 mechanism of the drug is currently unclear. Niclosamide reportedly reduces receptor-binding domain and dextran uptake while increasing that of transferrin, resulting in an enhanced density of transferrin endosomes (Prabhakara et al., 2021). In addition, the pH of both early and late endosomes changed from acidic to neutral after niclosamide treatment. These phenomena suggest that niclosamide inhibits viral entry into cells by affecting pH-dependent endocytosis pathways to hinder viral internalization (Prabhakara et al., 2021) (Figure 4D). A phase I trial showed that intranasal and inhalation administration of niclosamide exhibited dose-dependent pharmacokinetics, and was well-tolerated in healthy volunteers (Backer et al., 2021). Niclosamide add-on therapy was beneficial for reducing the time needed for recovery (5 vs. 7 days, *p* = 0.005), but it did not reduce mortality from SARS-CoV-2 infection (Abdulamir et al., 2021). Currently, eight relevant clinical trials on niclosamide have been registered.

Chlorpromazine is an FDA-approved drug for treating psychiatric disorders, nausea and vomiting during pregnancy, and cancer. *In vitro* experiments have shown that chlorpromazine has broad-spectrum antiviral activity. In Vero E6 and A549-ACE2 cell models, the IC_50_ value of chlorpromazine against SARS-CoV-2 was about 9–10 μM (Plaze et al., 2021). Inoue et al. found that chlorpromazine impedes the clathrin-mediated endocytosis pathway by inhibiting the relocalization of clathrin and adaptor protein 2 on the cell surface (Figure 4C), thus inhibiting SARS-CoV entry (Inoue et al., 2007). In addition, studies have shown that chlorpromazine has immunomodulatory effects and exerts antiviral effects by increasing IgM levels in the blood (Plaze et al., 2020). Therefore, chlorpromazine is likely to inhibit SARS-CoV-2 infection through the mechanisms mentioned above. Currently, there are two clinical trials registered for chlorpromazine to treat COVID-19, but recruitment has not yet been conducted.

Baricitinib is an oral JAK1/JAK2 inhibitor. In November 2020, it was authorized for emergency use in combination with remdesivir for the treatment of hospitalized COVID-19 patients requiring supplemental oxygen, non-invasive/invasive mechanical ventilation, or ECMO. Zhang X. et al. (2020) reported a possible mechanism of baricitinib against SARS-CoV-2. AAK1 and GAK are two key regulators of the ACE2 receptor, mediating clathrin-dependent endocytosis (Lu et al., 2020) (Figures 4C, E). Baricitinib has a high binding affinity for AAK1 and GAK, blocking SARS-CoV-2 entry into cells for replication and intracellular assembly. Baricitinib, on the other hand, is an ATP-competitive kinase inhibitor that selectively and effectively inhibits JAK1/JAK2. By inhibiting JAK1/JAK2, baricitinib inhibits the pro-inflammatory signals of various cytokines, such as IL-6, IL-12, and IL-23 (Keystone et al., 2015). In severe cases of COVID-19, the JAK-STAT signaling pathway is also involved in cytokine storms, which baricitinib may help suppress, thus preventing damage to the lungs and other organs (Zhang X. et al., 2020). In a randomized controlled trial, baricitinib plus standard of care treatment was associated with reduced mortality in hospitalized COVID-19 patients, and the 28-day all-cause mortality was 8% (*n* = 62) for baricitinib and 13% (*n* = 100) for placebo [HR 0.57 (95% CI 0.41–0.78); *p* = 0.0018] (Marconi et al., 2021).

#### 4.2.2 Inhibitors of TMPRSS2

Proxalutamide is an androgen receptor antagonist developed by Kintor Pharmaceutical Limited and was initially used to treat prostate cancer. According to related studies by Kintor, proxalutamide blocks the SARS-CoV-2 entry by inhibiting androgen receptor function and downregulating ACE2 and TMPRSS2 expression at the transcriptional level (Wu S. et al., 2020). ACE2 facilitates viral entry into host cells by binding to the S1 domain of the S protein. TMPRSS2 is located on the surface of host cells and is responsible for cleavage of viral S protein and ACE2, impeding subsequent membrane fusion during coronavirus entry. Activated androgen receptors induce the expression of TMPRSS2. Phase I/II clinical studies of proxalutamide for COVID-19 treatment have been completed, showing that proxalutamide can effectively accelerate viral clearance and reduce the time of clinical symptom alleviation in patients with mild to moderate COVID-19 (Cadegiani et al., 2021). However, the excellent trial data has also raised some questions about this anti-SARS-CoV-2 drug, plunging the drug into controversy. The most effective way to address the controversy is to obtain data from rigorous clinical trials. Several phase III clinical trials are currently underway in the United States and Brazil. The update of the phase III study (NCT04870606) of proxalutamide for COVID-19 was provided by Kintor Pharma, and the interim analysis of 348 patients enrolled in the trial showed that it did not meet the statistical criteria due to the small number of events (Kintor Pharma-B, 2021).

Dutasteride is a 5-alpha-reductase inhibitor that specifically binds to isoenzymes 1/2 of 5-alpha reductase to form a stable enzyme complex and inhibits the conversion of testosterone to 5-alpha-dihydrotestosterone. Dihydrotestosterone stimulates androgen receptor activity, and the activated androgen receptor regulates TMPRSS2 expression (Mollica et al., 2020). Therefore, it is likely that dutasteride affects viral entry into cells by reducing TMPRSS2 expression, and thus inhibits SARS-CoV-2 infection.

Camostat mesylate and nafamostat mesylate are structurally similar guanidinobenzoic acid derivatives. As cellular protease inhibitors, they can effectively suppress the activities of kallikrein, trypsin, thrombin, and TMPRSS2. Studies have shown that TMPRSS2 is a type II transmembrane serum protease widely expressed in epithelial cells that have been shown to facilitate the entry of a variety of viruses into cells, including influenza, SARS-CoV, and MERS-CoV (Breining et al., 2021). The TMPRSS2 inhibitors nafamostat mesylate and camostat mesylate effectively suppress SARS-CoV-2 activity *in vitro* (Hoffmann et al., 2020a; Wang M. et al., 2020). Several clinical trials of camostat mesylate for the treatment of COVID-19 have been carried out. The results of one randomized controlled trial showed no improvement in clinical symptoms and mortality in hospitalized COVID-19 patients (Gunst et al., 2021). A phase II clinical trial (NCT04623021) of nafamostat mesylate has been completed; however, the results are not yet available, and the phase III clinical trial (NCT04871646) for COVID-19 was conducted in South Korea in June. Camostat mesylate is rapidly hydrolyzed *in vivo* to its active metabolite 4-(4-guanidinobenzoyloxy) phenylacetic acid, with similar anti-SARS-CoV-2 activity (Hoffmann et al., 2021). Relevant model prediction studies have shown that camostat mesylate binds to all three pockets of TMPRSS2 (S1, catalytic triad domain, and hydrophobic patch). In contrast, bromhexine, another TMPRSS2 inhibitor, only binds to the hydrophobic patch domain (Breining et al., 2021). Furthermore, the hydrogen bond and van der Waals interaction with Asp^435^ of S1, Ser^441^ of the catalytic domain, and His^296^ of the hydrophobic patch were predicted. However, a report suggests that the anti-SARS-CoV-2 activity of camostat mesylate and its metabolites may not only result from the competitive inhibition of TMPRSS2 since covalent inhibition as substrates may also contribute to antiviral activity (Sun et al., 2020). Therefore, the molecular mechanism of camostat mesylate or GBPA on TMPRSS2 requires further study.

#### 4.2.3 Inhibitors of Dihydroorotate Dehydrogenase

Both PTC299 and teriflunomide are inhibitors of DHODH, a key enzyme in the pyrimidine *de novo* biosynthesis pathway, which SARS-CoV-2 can hijack for virus replication. DHODH inhibitors reportedly suppress the production of virus-induced inflammatory cytokines by regulating lymphocyte activation, exerting dual antiviral and anti-inflammatory effects (Xiong et al., 2020). In tissue culture, PTC299 exhibited potent anti-SARS-CoV-2 replication activity (EC_50_ of 2.0–31.6 μM). Consistent with reported DHODH requirements for inflammatory cytokine production, PTC299 effectively inhibited the production of IL-6, IL-17A, IL-17, and VEGF in tissue culture models (Luban et al., 2021). In Vero E6 cells, teriflunomide exhibited potent antiviral effects, with an EC_50_ of 26.06 μM. Similarly, teriflunomide and its active form leflunomide also exerted antiviral effects by limiting pyrimidine *de novo* synthesis and immunomodulatory effects (Xiong et al., 2020). Currently, there are three clinical trials of leflunomide against COVID-19 registered on ClinicalTrials.gov, as well as one clinical trial registration for PTC299 against COVID-19.

BAY 2402234 is a novel antitumor drug developed by Bayer, currently undergoing phase I clinical trials for myeloid malignancies. With regard to anti-SARS-CoV-2, various DHODH inhibitors have shown significant antiviral efficacy, especially BAY 2402234, which is currently the most effective small-molecule agent against SARS-CoV-2. BAY 2402234 can effectively reduce viral RNA load, with an IC_50_ of 7.0 nM in Vero E6 cells (Song, 2020). It has a good therapeutic potential for the treatment of SARS-CoV-2. The crystal structure of the DHODH protein with BAY 2402234 shows that the latter binds to the ubiquitin-binding site between the N-terminal helices, and predicts a non-classical hydrogen bond between the hydroxyl of Thr^63^ and the fluorine (hydrogen bond acceptor) of the terminal phenyl group (Christian et al., 2019). The triazolone group binds to the deep hydrophilic end of the pocket via hydrogen bonding with Gln^47^ andTyr^356^, the hydroxyl group interacts with Thr^360^ via a bound water molecule, and the compound has extensive hydrophobic interactions with several non-polar residues at the entrance of the binding site (Christian et al., 2019).

#### 4.2.4 Inhibitors of Selective Serotonin Reuptake

Fluvoxamine is a selective serotonin reuptake inhibitor that is FDA approved for the treatment of obsessive-compulsive disorder and depression. Several phase II clinical trials of fluvoxamine against COVID-19 have been registered. The TOGETHER randomized clinical trial (NCT04727424) was conducted to evaluate the effect of early fluvoxamine treatment and the results were published recently. In a per-protocol analysis of patients, acute treatment with fluvoxamine reduced the need for hospitalization among high-risk outpatients (RR 0.34, 95% BCI, 0.21–0.45) (Reis et al., 2022). Previous studies have shown that fluvoxamine has anti-inflammatory effects. In immune cells, fluvoxamine binds to S1R, leading to reduced production of immunomodulatory cytokines. In human endothelial cells, it reduces the expression level of inflammation-related genes (Rafiee et al., 2016; Rosen et al., 2019). Lenze et al. (2020) proposed that fluvoxamine may exert antiviral effects by affecting the S1R-IRE1 pathway or through its lysosomotropic properties. Kornhuber et al. (2021) showed that acid sphingomyelinase cleaves sphingomyelin into lipophilic ceramide, forming large gel-like rafts on the membrane, which SARS-CoV-2 can use to enter cells. Thus, fluvoxamine may exert its anti-SARS-CoV-2 effects by inhibiting acid sphingomyelinase.

#### 4.2.5 Inhibitors of eEF1A

Plitidepsin is a cyclic depsipeptide approved for the treatment of patients with refractory multiple myeloma in Australia. Several clinical trials have investigated its efficacy and safety as a candidate drug for COVID-19 treatment. White et al. (2021) showed that plitidepsin displays high anti-SARS-CoV-2 activity, with an IC_50_ value of 0.88 nM in a hACE2-HEK293T cell model, and the antiviral activity was achieved through eEF1A activity inhibition. eEF1A is involved in the enzymatic delivery of aminoacyl tRNA to ribosomes and aminoacylation-dependent tRNA export pathways during the mRNA translation of RNA viruses. Plitidepsin suppresses the translation of SARS-CoV-2 ORF1A and ORF1B by acting on eEF1A, resulting in reduced expression levels of polyproteins 1a and 1b, further decreasing the expression of NSPs such as RdRp (Wong and Damania, 2021). In addition, the drug inhibits the translation of subgenomic mRNAs, also leading to reduced expression levels of viral structural and auxiliary proteins.

#### 4.2.6 Inhibitors of IMP α/β1

Ivermectin is an FDA-approved antiparasitic agent with broad-spectrum antiviral activity. Ivermectin effectively inhibited SARS-CoV-2 infection in Vero-hSLAM cells (Frieman et al., 2007). However, the exact mechanism of action of the drug against SARS-CoV-2 is unclear. It may block the transport of viral proteins into the nucleus by targeting host IMP α/β1, thereby suppressing viral replication (Yang et al., 2020). Similarly, during SARS-CoV infection, IMP α/β1 has a potential role in the signal-dependent nucleocytoplasmic shutting of the virus nucleocapsid protein, potentially impacting host cell division (Caly et al., 2020). Besides, the viral ORF6 antagonizes the antiviral activity of the STAT1 transcription factor by sequestering IMP α/β1 on the ER/Golgi membrane (Frieman et al., 2007). In summary, ivermectin may exert anti-SARS-CoV-2 effects by inhibiting nuclear transport activity. To date, more than 60 registered clinical trials related to ivermectin for the treatment of COVID-19, only a few of which are randomized controlled trials, have reported mortality. Based on reported results, there is insufficient evidence on the efficacy of ivermectin against COVID-19; thus, the WHO does not recommend its use, except in clinical trials (Yavuz and Komsuoğlu Çelikyurt, 2021).

#### 4.2.7 Inhibitors of Phosphoinositide Kinase, FYVE-Type Zinc Finger Containing (PIKfyve)

Apilimod is a small-molecule inhibitor of PIKfyve. Apilimod was reported to have high anti-SARS-CoV-2 activity in A549-ACE2 and Vero cells, with IC_50_ values of 0.007 μM and <0.08 μM, respectively. The authors proposed that the antiviral activity could be ascribed to the inhibition of PIKfyve phosphorylation in SARS-CoV-2 infection (Bouhaddou et al., 2020). Bouhaddou et al. (2020) reported that PIKfyve is one of the major enzymes involved in PI (3,5) P2 synthesis during early endocytosis. Entry inhibition assay showed that apilimod effectively blocked the entry of SARS-CoV-2 pseudovirus particles into HEK293-hACE2 cells by inhibiting PIKfyve activity, suggesting that PIKfyve may be a potential target for viral entry into cells via endocytosis, and apilimod inhibits SARS-CoV-2 infection through this target. Kang et al. (2020) also suggested that apilimod interfered with endosome transport and prevented virus entry by inhibiting PIKfyve activity. However, it should be noted that apilimod also selectively inhibits IL-12 and IL-23 by inhibiting the phosphotransferase activity of PIKfyve (Cai et al., 2013). It has been reported that PIKfyve is an enzyme required for MHC class II trafficking to the cell surface, and apilimod-mediated inhibition of this process may reduce the expression of MHC class II and type I interferon. A similar phenomenon is observed during SARS-CoV-2 infection. Therefore, the use of apilimod may suppress the immune system in patients with COVID-19 (Baranov et al., 2020). AI Therapeutics, Inc. is conducting a phase II clinical trial (NCT04446377) to evaluate the efficacy of apilimod in patients with COVID-19.

#### 4.2.8 Antiviral Agents Targeting Multiple Life Cycle Stages

Nitazoxanide, a thiazolide drug approved by the FDA to treat protozoal and helminthic infections, exhibited broad-spectrum antiviral activity *in vitro* (Mahmoud et al., 2020). In the Vero E6 cell model, the EC_50_ value of nitazoxanide against SARS-CoV-2 was 2.12 μM (Wang M. et al., 2020). Regarding its antiviral mechanism of action, nitazoxanide may target multiple stages of the SARS-CoV-2 life cycle, including endocytosis and membrane fusion, viral genome synthesis, viral release, and the inflammatory response (Lokhande and Devarajan, 2021). Currently, there are about 30 related clinical trials registered on ClinicalTrials.gov. The results of a pilot trial (NCT04348409) showed that nitazoxanide treatment shortened hospitalization time (6.6 vs. 14.0 days, *p* = 0.021), accelerated viral clearance, and reduced inflammatory cytokines (Blum et al., 2021). A meta-analysis of randomized controlled trials showed that nitazoxanide could improve viral elimination (Zhang et al., 2021).

## 5 Discussion

As the COVID-19 pandemic continues, the research and development of anti-SARS-CoV-2 drugs remain of utmost importance. The drug discovery strategy applied in the early stages of the COVID-19 outbreak was an extensive screening of available agents for drug repurposing. With the subsequent and still ongoing elucidation of the function and structure of SARS-CoV-2 proteins, the screening of small molecules based on their targets will represent a more effective strategy to discover effective drugs against SARS-COV-2.

Targets include viral and host proteins involved with the viral life cycle. Although there are various therapeutic targets for the treatment against SARS-CoV-2, the major focus of small-molecule drug development is on RdRp and 3CLpro. RdRp acts as a crucial component in SARS-CoV-2 replication, and RdRp inhibitors have shown good efficacy against SARS-CoV-2 *in vitro* as well as in clinical trials, with examples including molnupiravir, AT-527, and favipiravir. 3CLpro, another enzyme essential for viral replication, is the main protease of SARS-CoV-2. Since there is no protease with a similar cleavage site to 3CLpro in humans, it is more likely to develop highly specific inhibitors with good safety and broad-spectrum anti-coronavirus activity, such as PF-07321332 and s-217622. There are relatively few studies on the anti-SARS-CoV-2 mechanism of action of drugs against host targets, probably because host intracellular pathways are complex and the mechanisms vary between indications, limiting the available relevant mechanistic studies. The current host targets of anti-SARS-CoV-2 clinical drugs mainly involve the factors from the viral endocytosis pathway, intracellular kinases (e.g., TMPRRS2, DHODH, PIKfyve), and cellular immune regulators. Considering that these targets exert multiple functions within the cell, drugs targeting them should be further characterized with respect to toxicity and timing of administration.

The elucidation of mechanisms may provide a theoretical basis for drug combinations. The widespread use of drugs usually leads to the development of drug resistance as a result of natural selection, and with the successive approval and widespread use of anti-SARS-CoV-2 small-molecule drugs, the emergence of drug-resistant strains will be one of the key concerns. One of the main strategies utilized for combating drug resistance is drug combinations, especially those with different mechanisms of action. Combining of drugs, such as molnupiravir and PF-07321332, to inhibit SARS-CoV-2 replication may theoretically lead to stronger effects and reduce the development of drug resistance. Indeed, whether this leads to better results needs to be verified in clinical trials. At the same time, whether the combination of drugs leads to stronger side effects is an issue that needs to be considered.

A key factor in efficacy of oral antivirals is administering early in the patient’s infection. This was the case in the clinical trials of PAXLOVID and molnupiravir, where the treatments were initiated within 3 and 5 days of symptom onset, respectively. Moreover, clinical studies of favipiravir for COVID-19 have also demonstrated better outcomes with early rather than late dosing in Japan (Shinkai et al., 2021). Another concern is whether the efficacy of oral antiviral drugs is compromised in patients who have developed breakthrough infections after vaccination. It's obvious that the emergence of Omicron, a new SARS-CoV-2 variant, has increased the risk of breakthrough infections. To our knowledge, the clinical efficacy of both PAXLOVID and molnupiravir have not been evaluated in clinical trials in vaccinated populations. A phase III clinical trial of favipiravir for COVID-19 showed that the median time to meet the primary endpoint was 2.9 days shorter in the subgroup of patients with IgA or IgG antibody-positive compared to the subgroup of patients with IgA or IgG antibody-negative (Shinkai et al., 2021). This indicates that oral antivirals may also show better efficacy in patients with breakthrough infections.

Discovering and clarifying the mechanism of action for candidate anti-SARS-CoV-2 drugs is a key point in developing broad-spectrum, target-specific drugs. The advance of structural biology, artificial intelligence, and bioinformatics has provided important tools for studying the mechanism of action and accelerating the development of antiviral drugs. Elucidating the mechanism of drug activity provides a theoretical basis for the development of highly effective and specific drugs, drug combinations, and multi-target drug development for the treatment of COVID-19.
